# p53 activation contributes to patulin-induced nephrotoxicity via modulation of reactive oxygen species generation

**DOI:** 10.1038/srep24455

**Published:** 2016-04-13

**Authors:** Huan Jin, Shutao Yin, Xinhua Song, Enxiang Zhang, Lihong Fan, Hongbo Hu

**Affiliations:** 1 Beijing Advanced Innovation Center for Food Nutrition and Human Health, Department of Nutrition and Health, College of Food Science and Nutritional Engineering, China Agricultural University, No17 Qinghua East Road, Haidian District, Beijing 100083, China; 2 College of Veterinary Medicine, China Agricultural University, No2 Yunamingyuan West Road, Haidian District, Beijing 100193, China

## Abstract

Patulin is a major mycotoxin found in fungal contaminated fruits and their derivative products. Previous studies showed that patulin was able to induce increase of reactive oxygen species (ROS) generation and oxidative stress was suggested to play a pivotal role in patulin-induced multiple toxic signaling. The objective of the present study was to investigate the functional role of p53 in patulin-induced oxidative stress. Our study demonstrated that higher levels of ROS generation and DNA damage were induced in wild-type p53 cell lines than that found in either knockdown or knockout p53 cell lines in response to patulin exposure, suggesting p53 activation contributed to patulin-induced ROS generation. Mechanistically, we revealed that the pro-oxidant role of p53 in response to patulin was attributed to its ability to suppress catalase activity through up-regulation of PIG3. Moreover, these *in vitro* findings were further validated in the p53 wild-type/knockout mouse model. To the best of our knowledge, this is the first report addressing the functional role of p53 in patulin-induced oxidative stress. The findings of the present study provided novel insights into understanding mechanisms behind oxidative stress in response to patulin exposure.

p53 is the first identified and the best known tumor suppressor that controls cell cycle checkpoints and apoptosis and DNA repair^1^. In addition to these traditional functions of p53, a growing body of evidence suggests that p53 plays an important role in the regulation of redox balance^2^. A number of studies have shown that p53 can exert pro-oxidant activity through regulation of its transcriptional targets such as p53-inducible genes (PIGs) or NCF2/p67phox, a cytosolic subunit of the NADPH oxidase enzyme complex^3^,^4^. In contrast, a number of other studies argue that p53 can function as antioxidant factor through regulation of several antioxidant proteins such as MnSOD (Manganese superoxide dismutase)^5^, GPx1 (glutathione peroxidase 1)^6^, Sestrins^7^, TIGAR (p53-induced glycolysis and apoptotic regulator)^8^ and GLS2 (Glutaminase 2)^9^. These controversial functions of p53 in the regulation of redox status are possibly associated with the conditions of the cells (non-stressed vs stressed).

Mycotoxins are secondary metabolites of fungi that can cause disease and death in human and animals. Patulin (the chemical structure of patulin are shown in Fig. S1C), a mycotoxin produced by a variety of molds, mainly Aspergillus and Penicillium, is commonly found in moldy fruits and their derivative products^10^. Exposure to patulin is reported to cause diverse toxic effects including dermal, immunological, neurological, gastrointestinal and nephrotoxic toxicities^10^,^11^,^12^. Mechanistically, previous studies have shown that patulin was able to induce oxidative DNA damage in multiple organ sites including kidney, liver, brain and urinary bladder^13^. Oxidative stress was suggested to play a pivotal role in patulin-induced multiple toxic signaling^14^,^15^,^16^. Consistent with DNA damage, p53 was activated in response to patulin exposure both *in vitro* and *in vivo*^17^,^18^,^19^. However, the functional role of p53 in the regulation of ROS generation by patulin has not been addressed. In the present study, using both cell culture and animal models, we demonstrated that p53 activation played a pro-oxidant role in patulin-induced oxidative stress through mechanisms involved in inhibition of anti-oxidant enzyme catalase activity by increase of PIG3 expression. To our knowledge, this is the first report addressing the functional role of p53 in patulin-induced oxidative stress.

## Results

### Inactivation of p53 decreased ROS generation in response to patulin exposure

**Figure 1:**
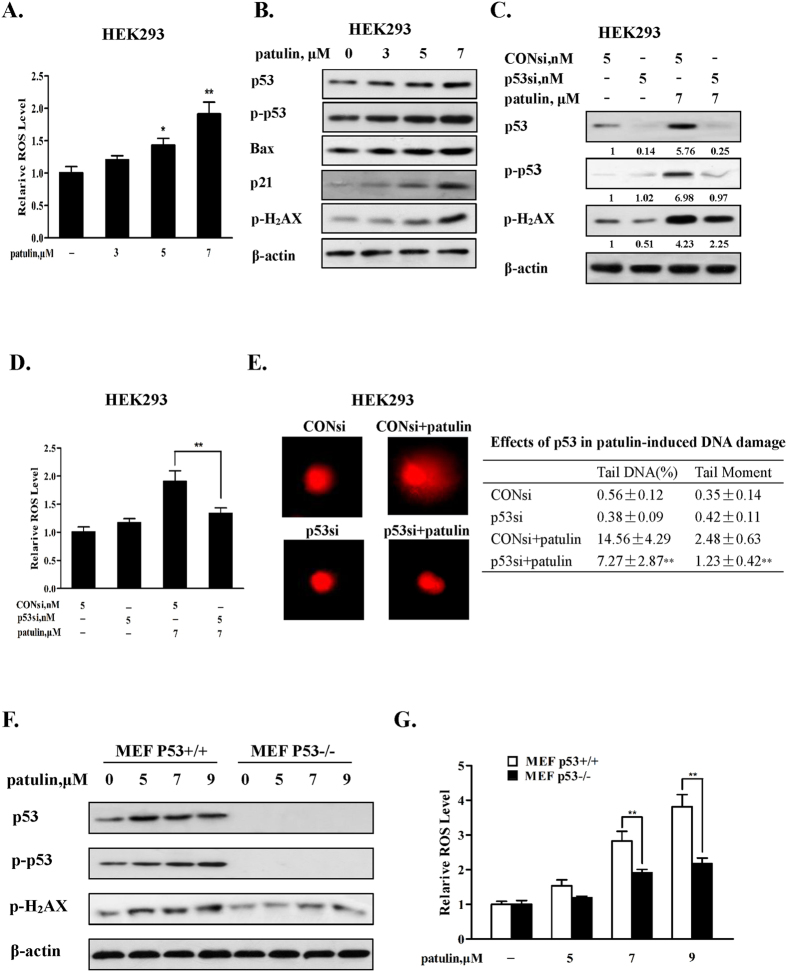
Inactivation of p53 decreased ROS generation in response to patulin exposure. (**A**). patulin induces ROS generation in a concentration-dependent manner in HEK293 cells. The cells were treated with various concentrations patulin for 24 h and intercellular ROS levels in response to patulin exposure were measured by flow cytometry after staining with H2DCFDA. (**B**). Effects of patulin on phosphorylation of H2AX and expression of p53, Bax and p21. The cells were treated with various concentrations patulin for 24 h and then the protein levels were analyzed by western blotting. (**C**). Effects of p53 inactivation by RNA interference on H2AX phosphorylation by patulin. The cells were transfected with p53 siRNA using INTERFER siRNA transfection agent. At 24 h post-transfection, the cells were treated with 7 μM patulin for 24 h and then H2AX phosphorylation was analyzed by western blotting. (**D**). Effects of p53 knockdown on ROS production by patulin. The cells were transfected with p53 siRNA using INTERFER siRNA transfection agent. At 24 h post-transfection, the cells were treated with 7 μM patulin for 24 h and intercellular ROS levels in response to patulin exposure were measured by flow cytometry after staining with H2DCFDA. (**E**). Effects of p53 inactivation on patulin-induced DNA damage. The cells were transfected with p53 siRNA using INTERFER siRNA transfection agent. At 24 h post-transfection, the cells were treated with 7 μM patulin for 24 h and DNA damage in response to patulin exposure were measured by comet assay. Effects of patulin on phosphorylation of H2AX and ROS levels in p53 wild type/knockout MEF cells. The cells were treated with various patulin concentrations for 12 h. H2AX phosphorylation (**F**) was analyzed by western blotting and ROS generation (**G**) was measured by flow cytometry after staining with H2DCFDA. n = 3, *P < 0.05, **P < 0.01. (The blots shown are representative of three independent experiments).

Previous studies have shown that patulin induced oxidative stress^13^,^14^,^15^,^16^ and p53 activation^17^,^18^,^19^. We first confirmed these findings in the cell line used in the present study. As shown in Fig. 1A, patulin treatment resulted in a concentration-dependent ROS generation accompanied by DNA damage (increase of DNA damage marker H2AX phsophorylation) and p53 activation evidenced by induction of its transcriptional targets Bax and p21 (Fig. 1B) in Human Embryonic Kidney (HEK) 293 cells. To determine the role of p53 activation in patulin-induced ROS generation, we examined the influences of p53 inactivation by RNAi approach on the levels of ROS. As shown in Fig. 1C, p53 was efficiently inhibited by p53 siRNA. Under such condition, patulin-induced ROS was significantly decreased compared with that of control siRNA/patulin treatment (Fig. 1D). Consistent with the decreased ROS generation, DNA damage marker H2AX phosphorylation, % tail DNA and tail moment induced by patulin were dramatically ameliorated when p53 was silenced (Fig. 1C,E). Moreover, the functional role of p53 in patulin-induced ROS was further assessed in p53 knockout mouse embryonic fibroblast (MEF) cells. Patulin caused a concentration-dependent increase of both phospho- and total p53 in MEF cells (Fig. 1F). In line with the p53 activation, a significant higher level of ROS was observed in p53 wild type MEF cells than that found in p53 knockout MEF cells (Fig. 1G). Consistent with the levels of ROS, a decreased DNA damage marker H2AX phosphorylation induced by patulin was detected in p53 knockout MEF cells compared with that in p53 wild type MEF cells (Fig. 1F). Taken together, these results clearly indicated that p53 activation functioned as a pro-oxidant mediator to facilitate ROS generation in response to patulin exposure in the cell lines tested.

### The pro-oxidant function of p53 in patulin-induced oxidative stress was associated with inhibition of catalase

**Figure 2:**
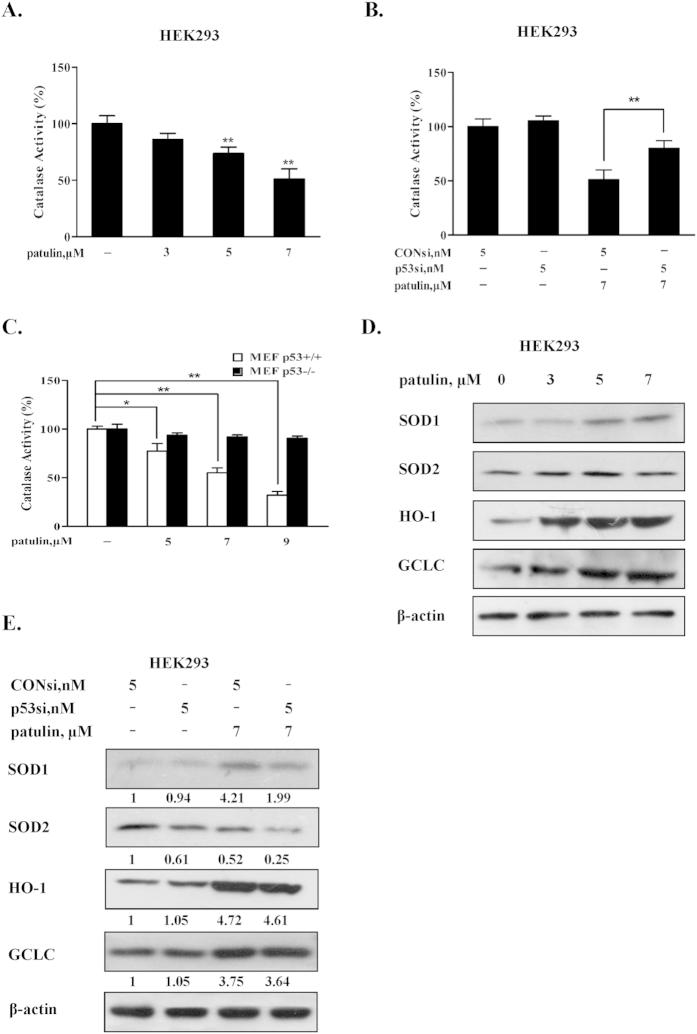
The pro-oxidant function of p53 in patulin-induced oxidative stress was associated with inhibition of catalase. (**A**). Patulin repressed catalase activity in HEK293 cells. The cells were treated with various concentrations patulin for 24 h and then the catalase activity was measured using catalase activity assay kit. (**B**). Influences of p53 knockdown on catalase activity by patulin. The cells were transfected with p53 siRNA using INTERFER siRNA transfection agent. At 24 h post-transfection, the cells were treated with 7 μM patulin for 24 h and then the catalase activity was measured by catalase activity assay kit. (**C**). Inhibitory effects of patulin on catalase activity in p53 knockout/wild type MEF cells. The cells were treated with various patulin concentrations for 12 h and then the catalase activity was measured by catalase activity assay kit. (**D**). Effects of patulin on superoxide dismutase (SOD) and Nuclear erythroid 2-related factor 2 (Nrf2). The cells were treated with various concentrations patulin for 24 h and then expression of SOD1, SOD2, HO-1 and GCLC were analyzed by western blotting. (**E**). Influences of p53 knockdown on antioxidant enzymes SOD and Nrf2 in response to patulin. The cells were transfected with p53 siRNA using INTERFER siRNA transfection agent. At 24 h post-transfection, the cells were treated with 7 μM patulin for 24 h and then expression of SOD1, SOD2, HO-1 and GCLC were analyzed by western blotting. n = 3, *P < 0.05, **P < 0.01. (The blots shown are representative of three independent experiments).

It has been shown that ROS generation by patulin was associated with decrease of catalase activity^11^,^15^. We first confirmed these data in the cell line used in the present study. As shown in Fig. 2A, exposure to patulin led to a concentration-dependent suppression of catalase activity in HEK293 cells. We next asked whether the pro-oxidant function of p53 was attributed to its ability to suppress catalase activity. To address this issue, p53 was inactivated by knockdown approach in HEK293 cells. Under such condition, catalase activity was measured using a catalase activity assay kit. As shown in Fig. 2B, when p53 was silenced, the inhibitory effects of patulin on catalase activity were significantly attenuated in HEK293 cells. In addition, similar results were also observed in p53 wild type/knockout MEF cell model systems (Fig. 2C). These results suggested that inhibition of catalase activity was involved in the pro-oxidant function of p53 in both cell lines tested in response to patulin exposure. We also investigated the possible involvement of superoxide dismutase (SOD) and Nuclear erythroid 2-related factor 2 (Nrf2), the two important oxidative stress related enzymes, in patulin-induced pro-oxidant action of p53. As shown in Fig. 2D, treatment with patulin resulted in an obvious increase of SOD1 and activation of Nrf2 evidenced by increased its two transcriptional targets Heme oxygenase 1(HO-1) and glutamate-cysteine ligase catalytic subunit (GCLC). When p53 was inhibited by its siRNA, both SOD1 and SOD2 were decreased without obvious changes of HO-1 and GCLC in comparison with consi/patulin treatment (Fig. 2E), suggesting that superoxide dismutase and Nrf2 unlikely contributed to the pro-oxidant function of p53 in response to patulin exposure.

### Induction of PIG3 was required for the pro-oxidant function of p53 in patulin-induced oxidative stress

**Figure 3:**
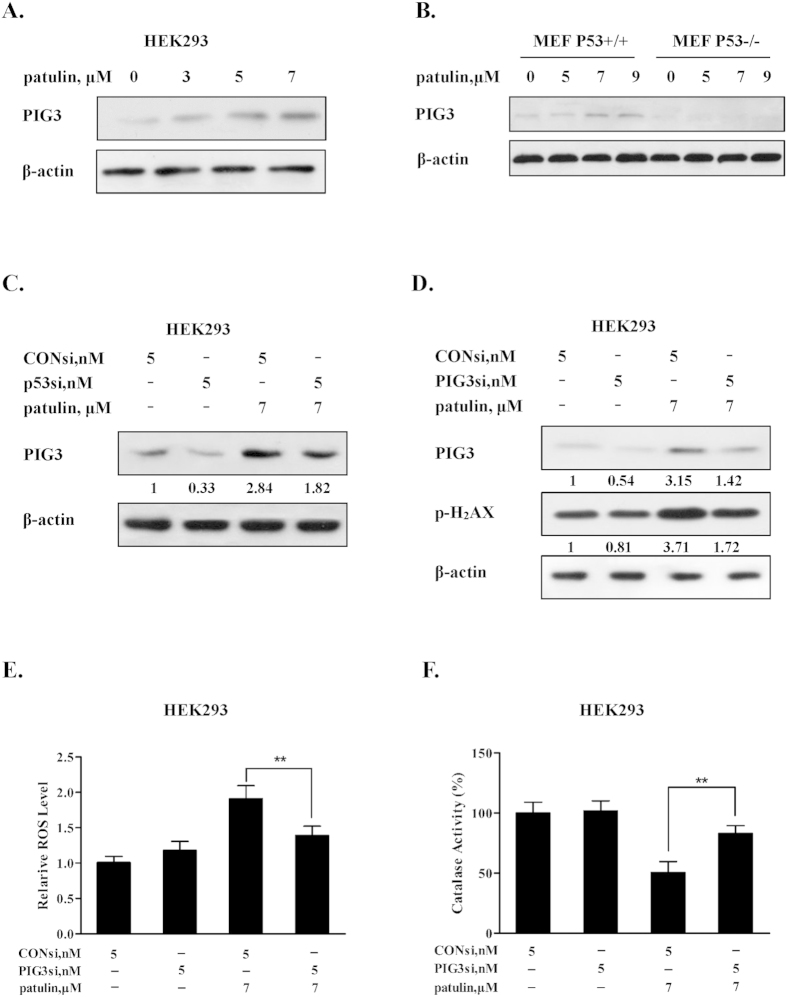
Induction of PIG3 was required for the pro-oxidant function of p53 in patulin-induced oxidative stress. (**A**). Effects of patulin treatment on PIG3 expression in HEK293 cells. The cells were treated with various concentrations patulin for 24 h. The protein level of PIG3 was analyzed by western blotting. (**B**). Effects of patulin treatment on PIG3 expression in p53 wild type/knockout MEF cells. The cells were treated with various patulin concentrations for 12 h and then PIG3 expression was measured by western blotting. (**C**). Effects of p53 knockdown on PIG3 expression by patulin. The cells were transfected with p53 siRNA using INTERFER siRNA transfection agent. At 24 h post-transfection, the cells were treated with 7 μM patulin for 24 h and then PIG3 expression was analyzed by western blotting. (**D**). Effects of PIG3 knockdown on H2AX phosphorylation by patulin. The cells were transfected with PIG3 siRNA using INTERFER siRNA transfection agent. At 24 h post-transfection, the cells were treated with 7 μM patulin for 24 h and then H2AX phosphorylation was measured by western blotting. (**E,F**). Effects of PIG3 inactivation on patulin induced ROS generation and inhibitory effects of catalase activity. The cells were transfected with PIG3 siRNA using INTERFER siRNA transfection agent. At 24 h post-transfection, the cells were treated with 7 μM patulin for 24 h. ROS levels were measured by flow cytometry after staining with H2DCFDA (**E**) and catalase activity was analyzed by catalase activity assay kit (**F**). n = 3, *P < 0.05, **P < 0.01. (The blots shown are representative of three independent experiments).

PIG3 (p53-inducible gene 3), a transcriptional target of p53, has been reported to play a role in the pro-oxidant activity of p53 in some model systems^3^,^20^,^21^. We then asked if up-regulation of PIG3 contributed to patulin-induced p53-mediated ROS generation. The changes of PIG3 expression in response to patulin exposure were analyzed by western blotting and the results are shown in Fig. 3A,B. As expected, exposure to patulin caused a concentration-dependent up-regulation of PIG3 in both HEK293 and p53 wt MEF cells, further supporting transcriptional activation of p53 in response to patulin exposure in these cell lines. Moreover, we confirmed that up-regulation of PIG3 was indeed due to p53 activation by the evidence that knockdown of p53 led to a decreased PIG3 expression in HEK293 cells (Fig. 3C), whereas knockout of p53 resulted in abolishment of PIG3 induction in MEF cells (Fig. 3B). To examine the role of PIG3 induction in patulin-induced ROS generation, we measured the influences of PIG3 inhibition by RNAi on the levels of ROS generation. As shown in Fig. 3D, PIG3 was efficiently suppressed by its siRNA. Under such condition, DNA damage marker H2AX phosphorylation induced by patulin exposure was inhibited partially. Consistent with the decreased DNA damage, the ROS generation by patulin was suppressed under PIG3 silencing condition (Fig. 3E). In line with the decreased ROS generation, the inhibition of catalase activity by patulin was significantly attenuated when PIG3 was inactivated by RNAi (Fig. 3F). Together, the results suggested that p53-dependent up-regulation of PIG3 contributed to ROS generation in response to patulin exposure.

### ROS generation preceded p53 activation in response to patulin exposure

**Figure 4:**
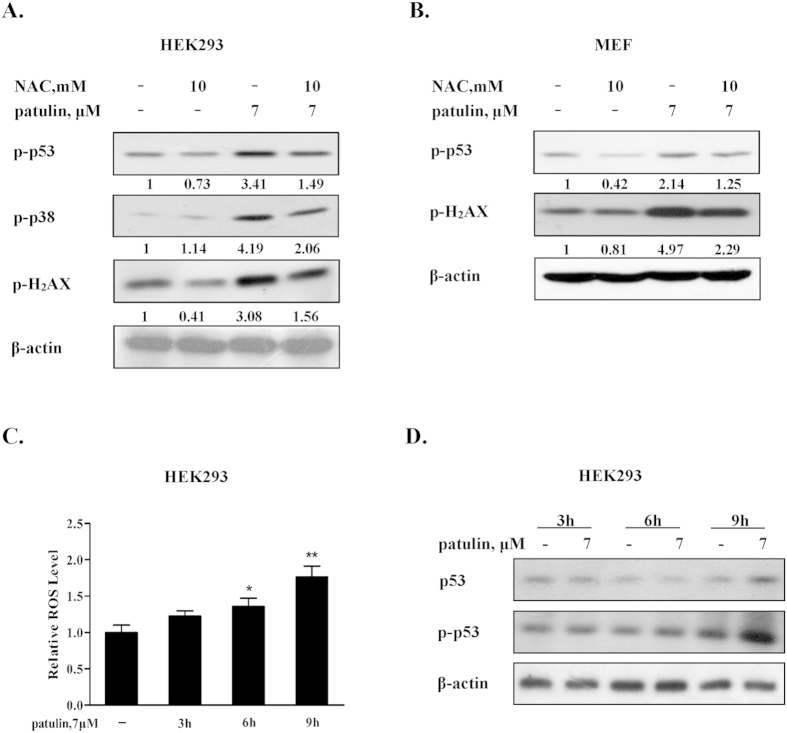
ROS generation preceded p53 activation in response to patulin exposure. (**A**). Effects of ROS inhibition by NAC on p53, p38 and H2AX phosphorylation by patulin in HEK293 cells. The cells were treated with patulin in the presence or absence of NAC for 24 h and then the p53 and H2AX phosphorylation were analyzed by western blotting. (**B**). Effects of ROS inhibition by NAC on p53 and H2AX phosphorylation by patulin in p53 wide type MEF cells. The cells were treated with patulin in the presence or absence of NAC for 12 h and then p53 and H2AX phosphorylation were analyzed by western blotting. (**C**). Time-course of ROS generation by patulin in HEK293 cells. The cells were treated with patulin for the indicated times and intercellular ROS levels in response to patulin exposure were measured by flow cytometry following staining with H2DCFDA. (**D**). Time-course analysis of total p53 and p53 phosphorylation. The cells were treated with patulin for the indicated time and then p53 and p53 phosphorylation were assessed by western blotting. n = 3, *P < 0.05, **P < 0.01. (The blots shown are representative of three independent experiments).

Based on the literatures, the consequence of oxidative stress is thought to be DNA damage which in turn led to p53 activation in certain conditions^22^. To determine the role of ROS generation in patulin-induced DNA damage and p53 activation, we tested effects of ROS inhibition by N-acetyl-1-cysteine (NAC), a precursor of intracellular glutathione synthesis and ROS scavenger, on H2AX, p53 and p38 phosphorylation in response to patulin. As shown Fig. 4A,B, patulin-induced phosphorylation of H2AX, p38 and p53 was obviously attenuated in the presence of NAC in both HEK293 and p53 wt MEF cells. Blockade of ROS led to inhibition of p53 activation, whereas inactivation of p53 resulted in decreased ROS generation in response to patulin exposure (Fig. 1D,G), suggesting a feedback loop existed between ROS generation and p53 activation. To determine which one was the primary event, we carried out a time kinetic study assessing dynamic changes of ROS generation and p53 phosphorylation. As shown in Fig. 4C,D, exposure to patulin induced a rapid ROS generation and a significant increase of ROS level was detected as early as after 6 h of patulin exposure, whereas the increased p53 phosphorylation was observed at 9 h of patulin treatment. These results indicated that p53 activation was initiated by ROS generation and activation of p53 in turn promoted ROS production through regulation of PIG3/catalase axis.

### p53 exerted pro-apoptotic activity through a transcriptional mechanism

**Figure 5:**
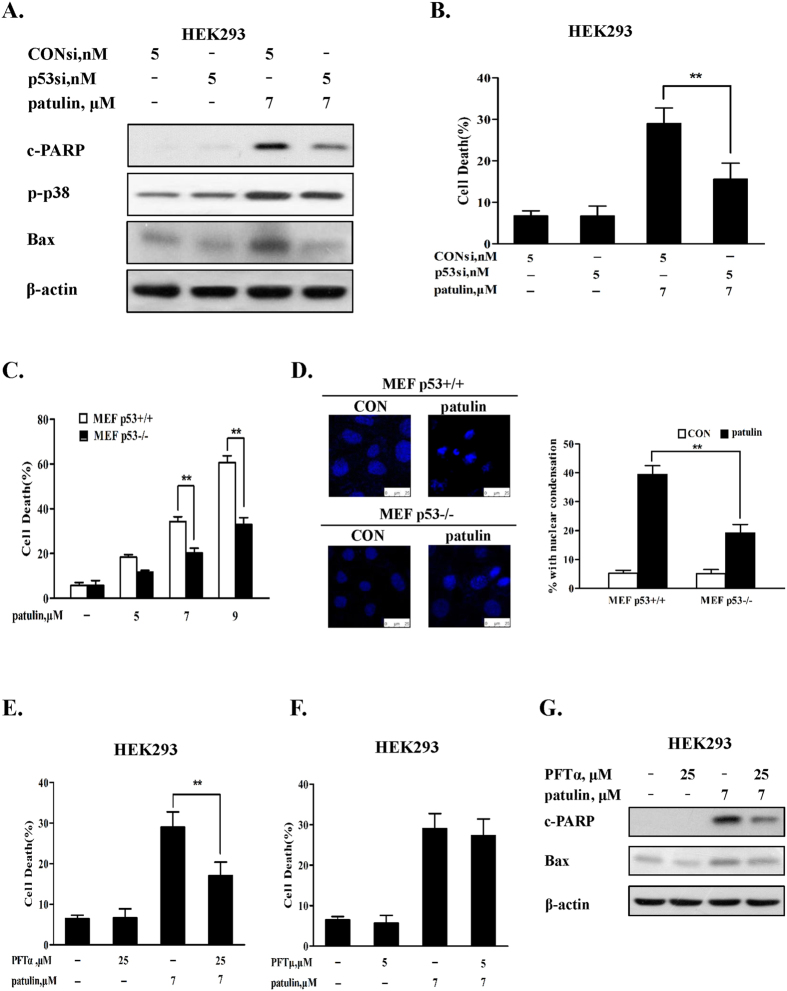
p53 exerted pro-apoptotic activity through a transcriptional mechanism. (**A**). Effects of p53 inhibition by RNAi on patulin-induced Bax up-regulations, p38 phosphorylation and PARP1 cleavages. The cells were transfected with p53 siRNA using INTERFER siRNA transfection agent. At 24 h post-transfection, the cells were treated with 7 μM patulin for 24 h and then Bax, p38 phosphorylation and PARP cleavages were analyzed by western blotting. (**B**). Effects of p53 knockdown on patulin-induced apoptosis. The cells were transfected with p53 siRNA using INTERFER siRNA transfection agent. At 24 h post-transfection, the cells were treated with 7 μM patulin for 30 h and then apoptosis was measured by Annexin V staining. (**C**). Patulin induced apoptosis in p53 knockout/wild type MEF cells. The cells were treated with various patulin concentrations for 18 h and then apoptosis was measured by Annexin V staining. (**D**). PAT induced nuclear morphological changes in p53 knockout/wild type MEF cells. The cells were treated with 7 μM patulin for 12 h and then the nuclei were stained with DAPI. (**E**). Effects of p53 inhibitor pifithrin-α on patulin-induced apoptosis measured by Annexin V staining. (**F**). Effects of p53 inhibitor pifithrin-μ on patulin-induced apoptosis measured by Annexin V staining. (**G**). Effects of p53 inhibitor pifithrin-α on patulin-induced Bax up-regulations. The cells were treated with patulin in the presence or absence of pifithrin-α for 24 h and then Bax expression was analyzed by western blotting. n = 3, *P < 0.05, **P < 0.01. (The blots shown are representative of three independent experiments).

Having established the pro-oxidant role of p53 activation in patulin-induced oxidative stress, we next investigated the role of p53 in patulin-induced apoptosis. As shown in Fig. 5A, when p53 was inactivated by RNAi, Bax up-regulation, p38 phosphorylation and PARP cleavages induced by patulin were significantly decreased in HEK293 cells. Accordingly, apoptosis induction by patulin was also decreased under the condition of p53 inactivation in HEK293 cells (Fig. 5B). In addition, a significant decreased apoptosis induction result was also observed in p53 knockout MEF cells in comparison with that found in p53 wt MEF cells measured by annexin v staining (Fig. 5C) and nuclear DAPI staining (Fig. 5D). These results suggested that p53-mediated Bax activation might contribute to apoptosis induction by patulin. We further validated the pro-apoptotic role of p53 by measuring apoptosis in the presence of pifithrin-alpha^23^ or pifithrin-mu^24^,^25^, the two chemical inhibitors of p53 that can block p53-dependent transcriptional activation and inhibit transcriptional-independent p53 binding to mitochondria respectively. As shown in Fig. 5E,F, treatment with pifithrin-alpha led to a significant suppression of patulin-induced apoptosis, whereas pifithrin-mu failed to offer any protective effect on apoptosis induction by patulin. In support the protective effect of pifithrin-alpha, Bax induction by patulin was also blocked in the presence of pifithrin-alpha (Fig. 5G) but not in the presence of pifithrin-mu (data not shown). These results suggested that p53 activation contributed to apoptosis in response to patulin through its transcriptional mechanism.

### P53 activation promoted patulin-induced oxidative stress *in vivo*

**Figure 6:**
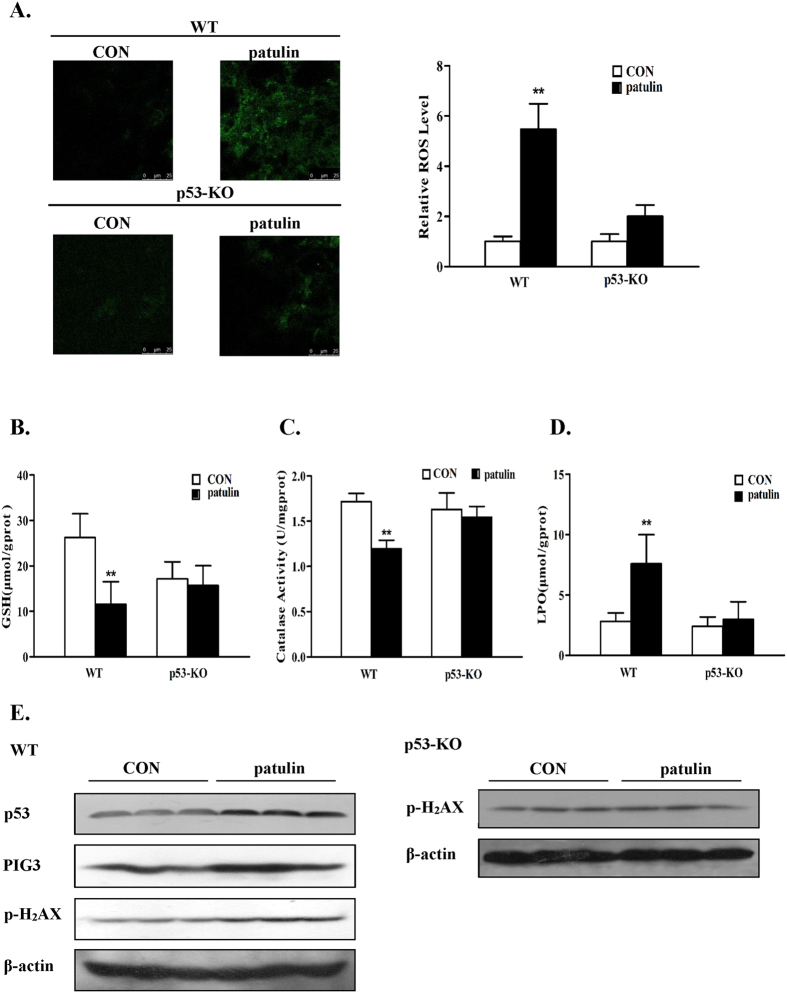
p53 activation promoted patulin-induced oxidative stress *in vivo*. (**A**). ROS levels were measured in p53wide type/knockout mice in response to patulin exposure. Frozen kidney sections were stained with 10 μM H2DCFDA for 30 min at 37 °C. Cells staining positively for the oxidized dye were identified by confocal microscopy. (**B-D**). Oxidative injury caused by patulin in p53 wide type/knockout mice. Kidney damage was assessed by measuring GSH (**B**), CAT (**C**) and LPO (**D**). (**E**). Western blotting analysis of p53, PIG3 and H2AX phosphorylation in kidney tissues. **P < 0.01. (The blots shown are representative of three independent experiments).

The above data have established a pro-oxidant function of p53 in response to patulin exposure in the cell culture model. We further validated these *in vitro* findings in a homozygous p53 knockout mouse model. To know the kinetic process of patulin-induced oxidative stress *in vivo*, we carried out a time-course experiment using p53 wild-type mouse model. Patulin was given by i.p injection for 1, 3, 6 or 12 h and then the samples were collected for analysis of the oxidative stress biomarkers. As shown in Fig. S1A, patulin caused a rapid (as early as 3 h) inhibition of glutathione and catalase activity and increase of lipid oxidation. These effects were gradually decreased starting from 12 h of exposure. Based on the time-course results, 3 h exposure design was chosen to investigate the functional role of p53 in patulin-induced oxidative stress *in vivo*. As shown in Fig. 6A, Fluorescence microscope observation showed that patulin caused a significant elevated DCF fluorescence intensity in the kidney tissues of p53 WT mice. In contrast, the DCF fluorescence intensity by patulin was dramatically attenuated in the kidney tissues of p53 knockout mice, supporting p53-dependent ROS generation by patulin *in vivo*. Consistent with the ROS data, the contents of glutathione (GSH) (Fig. 6B) and catalase activity (Fig. 6C) were significantly decreased in response to patulin exposure, accompanied by increased lipid oxidation (LPO) (Fig. 6D), in p53 WT mice, whereas such changes were not significant in p53 KO mice at the experimental condition. Accordingly, the induction of p53 and its target PIG3 was observed in patulin-treated p53 WT mice, which was paralleled with the increased H2AX phosphorylation. PIG3 was not detectable in p53 KO mice, which was probably due to p53 deficiency, whereas a slightly increased H2AX phosphorylation in response to patulin exposure was found in p53 KO mice (Fig. 6E). We also analyzed the histopathological changes by patulin in both p53 wild-type and knockout mice and the results are shown in Fig. S1B. No obvious pathological changes were found in the kidney tissues of both p53 wild-type (1, 3, 6 or 12 h treatment) and knockout mice (3 h treatment), suggesting continuous treatment and persistent oxidative stress may be required for a detectable pathological damage. Taken together, these data clearly supported a pro-oxidant role of p53 in response to patulin in the animal model.

## Discussion

Induction of oxidative stress is considered to be one of the major mechanisms behind patulin-induced multiple toxic effects including nephrotoxicity^13^,^14^,^15^,^16^. A better understanding of the molecular mechanisms by which patulin causes oxidative stress is needed for a better management of patulin-caused toxicities. Our present study uncovered for the first time that p53-dependent ROS generation mechanism was involved in patulin-induced oxidative stress in kidney cells both *in vitro* and *in vivo*.

The implication of p53 in the regulation of redox status still remains controversial^2^. Activation of p53 functions either as pro-oxidant or anti-oxidant signaling. In the present study, we first confirmed p53 activation in response to patulin exposure by measuring status of p53 phosphorylation and its transcriptional targets Bax and p21 expression (Fig. 1B). We then examined the functional role of p53 activation in ROS generation and DNA damage using both cell culture and animal models by genetic manipulation of p53 gene. In cell culture models, knockdown of p53 resulted in a significantly decreased ROS generation and DNA damage induced by patulin in HEK293 cells (Fig. 1). These outcomes were further confirmed in p53 knockout MEF cells. In the follow-up animal model, the results showed that a number of oxidative stress markers in kidney tissues induced by patulin were ameliorated in p53-KO mice compared with that found in p53-WT mice (Fig. 6). In addition, our data supported a positive feedback loop existed between ROS and p53 activation evidenced by inhibition of ROS leading to suppression of p53 activation and vice versa. The time-course study supported ROS generation was the primary event which triggered p53 activation and activation of p53 in turn augmented ROS production through a feedback loop (Fig. 4). It has been shown that patulin has a strong affinity for sulfhydryl groups^26^. We speculated that the rapid ROS generation by patulin was likely due to its electrophilic attack of the enzymes containing sulfhydryl group such as glutathione. This notion was supported by the evidence that exposure to patulin led to a significant inhibition of glutathione activity^27^. The findings of the present study therefore supported a pro-oxidant role of p53 activation in patulin-induced nephrotoxicity.

Having established the critical role of p53 activation in the oxidative stress induced by patulin, we next investigated the mechanisms by which p53 exerted pro-oxidant function. It has been shown that PIG3, a p53-regulated gene, cooperating with p53, can suppress catalase activity through direct interaction in certain conditions^20^ and decreased catalase activity has been reported to contribute to ROS generation induced by patulin both *in vitro*^15^ and *in vivo*^11^. We therefore assessed the role of PIG3/catalase axis in p53-dependent ROS generation. Our data showed that exposure to patulin induced a concentration-dependent p53 activation, which was paralleled with up-regulation of PIG3 (Fig. 3) and suppression of catalase activation (Fig. 2) in both HEK293 and MEF cells. Inactivation of p53 by either knockdown or knockout approach resulted in a decreased PIG3 expression, whereas an ameliorated catalase activity inhibition by patulin was observed under the condition of p53 deficiency. Furthermore, silencing of PIG3 by RNA interference led to a significant recover of catalase activity, which was accompanied by decreased ROS generation (Fig. 3). Consistent with the above *in vitro* findings, higher level of ROS and lower level of catalase activity in response to patulin exposure were detected in p53-WT mice than that found in p53-KO mice, which were consistent with PIG3 expression (Fig. 6). In kidney tissues of p53-KO mice, relative lower GSH level (Fig. 6B) and higher H2AX phosphorylation (Fig. 6E) were observed compared with p53-WT mice. The possible reason is that the basal p53 generally functions as antioxidant factor through regulation of several antioxidant proteins including glutathione. Inhibition of basal p53 may cause increase basal ROS level, which in turn led to increased H2AX phosphorylation. Taken together, our results clearly suggested that PIG3-catalase axis were involved in pro-oxidant function of p53 in response to patulin exposure.

p53 activation can exert either pro-apoptotic or pro-survival function^28^,^29^. Our present study showed that a significantly decreased cell death induction was detected in both p53 knockdown HEK293 human kidney cells and p53 knockout MEF cells than that found in their respective p53 wild-type cells. These findings indicated that p53-dependent cell death induction was involved in patulin-induced cytotoxicity. It has been shown that p53 activation can trigger apoptosis through either transcriptional-dependent or -independent mechanisms. For transcriptional pathway, p53 translocates into the nuclei and functions as transcriptional activator to activate its transcriptional targets such as pro-apoptotic proteins Bax, puma and NOXA^30^. For transcriptional-independent pathway, p53 translocates into the mitochondria, leading to activation of mitochondrial pathway through forming complexes with the anti-apoptotic Bcl-2 family proteins^31^. Alternatively, cytosolic p53 can directly trigger Bax activation and apoptosis^32^. Our data showed that exposure to patulin caused up-regulation of Bax and p21, two transcriptional targets of p53, but no p53 mitochondrial translocation was observed (data not shown), suggesting p53 transcriptional mechanism might be involved in patulin-induced p53-dependent cell death. This notion was supported by the experiment in which pifithrin alpha (α), a transcriptional inhibitor of p53^23^, significantly inhibited patulin-induced Bax expression and apoptosis induction, but such protective effect was not found with pifithrin-mu which was considered to be a specific inhibitor of p53 transcriptional-independent pathway^24^,^25^. In addition, a previous study has established the critical role of p38 activation in patulin-induced apoptosis^33^, whereas our present study revealed that p38 activation by patulin was partially p53-dependent (Fig. 5A). Given the established pro-oxidant role of p53 in the present study, we speculated that p53-dependent p38 activation was attributed to its ability to trigger ROS generation. This hypothesis was supported by the data that inhibition of ROS by anti-oxidant NAC led to a significant decreased p38 activation induced by patulin (Fig. 4A). The findings of the present study suggested that the pro-death function of p53 in response to patulin was associated with its transcriptional activation of pro-apoptotic proteins and activation of ROS-p38 axis.

**Figure 7:**
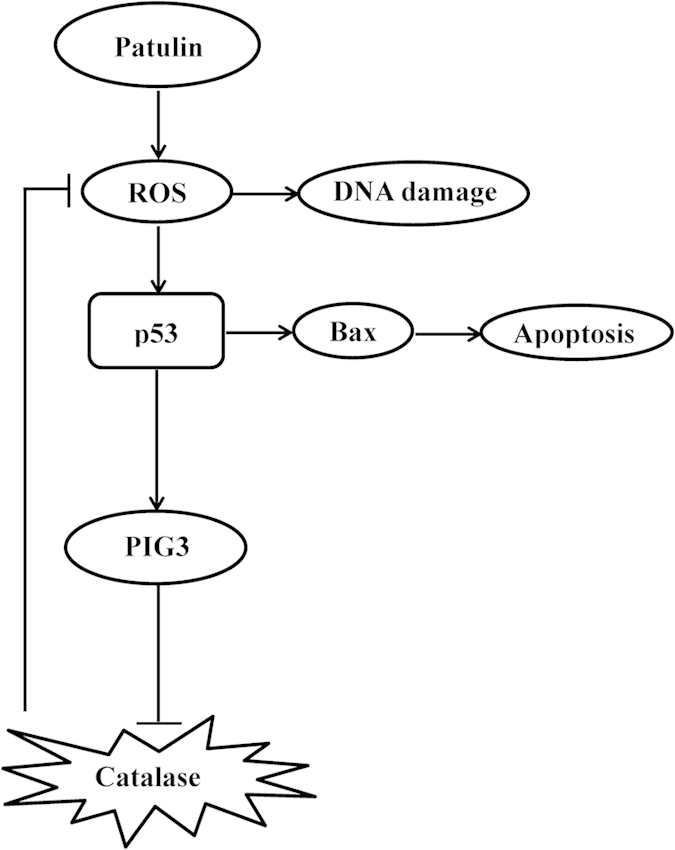
Signaling pathways underlying the pro-oxidant function of p53 in patulin-induced oxidative stress. Exposure to patulin induced ROS generation, DNA damage and p53 activation. Activation of p53 promoted patulin-induced ROS generation through a mechanism of PIG3-dependent inactivation of catalase. p53 activation contributed to patulin-induced apoptosis through mechanisms involved in its transcriptional-dependent activation of mitochondrial pathway.

In summary (Fig. 7), exposure to patulin induced ROS generation, DNA damage and p53 activation. Activation of p53 promoted patulin-induced ROS generation through a mechanism of PIG3-dependent inactivation of catalase. p53 activation contributed to patulin-induced apoptosis through mechanisms involved in its transcriptional-dependent activation of mitochondrial pathway and augmentation of ROS-mediated p38 activation.

## Materials and Methods

### Chemicals and reagents

Patulin, N-acetyl-1-cysteine (NAC), H_2_DCFDA, Pifithrin-μ (PFT-μ), Pifithrin-α (PFT-α), propidium iodide (PI) and 4′,6′-Diamidino-2-phenylindole (DAPI) were purchased from Sigma-Aldrich (St. Louis, MO, USA). Antibodies specific for total p53, phospho-p53 (ser15), p21, phsopho-p38, Bax, Phospho-Histone H2A.X (Ser139), cleaved poly (ADP-ribose) polymerase (PARP; p89), HO-1 and β-actin were purchased from Cell Signaling Technology (Beverly, MA,USA). Antibody for GCLC was purchased from Abcam (Cambridge, MA, USA). Antibodies specific for PIG3, SOD1 and SOD2 were purchased from Bioworld Technology (Minneapolis, MN, USA).

### Cell culture and treatments

HEK293 and MEF cells (generously provided by Professor Jiahuai Han, School of Life Sciences, Xiamen University, Xiamen, China) were grown in Dulbecco’s Modification of Eagle’s Medium (DMEM) (Thermo, Waltham, MA, USA; SH30022.01B) supplemented with 10% fetal bovine serum without antibiotics. When cells density reached about 50–60%confluence, the medium was changed with fresh medium containing patulin and/or other agents.

### Apoptosis evaluation

Apoptosis was assessed by three methods. The first one was Annexin V staining of externalized phosphatidylserine in apoptotic cells by flow cytometry using Annexin V/FITC staining kit from MBL International. The second method was immunoblot analysis of PARP1 cleavage. The third one was nuclear DAPI staining.

### Measurement of ROS

Generation of intercellular ROS was measured by flow cytometry following staining with H_2_DCFDA. H_2_DCFDA is a reduced form of 2,7-dichlorofluorescein. Oxidation by hydrogen peroxide can be detected by monitoring the increase in green fluorescence with a flow cytometer. At 30 min before harvest, H_2_DCFDA was added to the medium to a concentration of 5 μmol/L. The fluorescence was measured using a Becton Dickinson flow cytometer.

### Comet assay

After the treatment, the cells were collected and suspended in PBS. A mixture of 50 μL cell suspension with 50 μL 1.0% low melting agarose was added onto 0.5% agarose precoated frosted slides. After solidification on ice for 10 min, slides were lyzed in lysis buffer (2.5 M NaCl, 100 mM Na_2_EDTA•2H_2_O, 10 mM Tris, 1% Sodium lauroyl sarcosin, pH 10, containing 1% Triton X-100) for 1 h at 4 °C. Then the slides were put into the electrophoresis solution (1 M Na_2_EDTA•2H_2_O, 0.3 M NaOH, pH >13), and allowed for 20 min to unwind the nuclear DNA. Electrophoresis was conducted subsequently for 20 min at 300 mA, 25 V. After electrophoresis, the slides were neutralized twice for 15 min in neutralization buffer (0.4 M Tris–HCl, pH 7.5). Before analysis, slides were stained with propidium iodide (PI). Comet images were examined by fluorescence microscope (Nikon ECLIPSE E400, Japan) at 200 × magnification. Quantification of DNA damage was analyzed by software program (CaspLab-Comet Assay Software Project, version 1.2.3b1). The tail moment and the percentage of DNA in the tail (% Tail DNA) were used as DNA damage indicators.

### Western blotting

The cell was lysed with ice-cold RIPA (radioimmunoprecipitation assay) buffer. Equal amount of proteins of the samples was loaded onto the gel. After electrophoretic separation, the proteins were transferred to a nitrocellulose membrane. The membrane was subsequently incubated with primary antibodies followed by recognition with corresponsive secondary antibody. The immunoreactive bands were detected using enhanced chemiluminescence (Fisher/Pierce, Rockford, IL, USA) and recorded on an X-ray film (Eastman Kodak Company, Rochester, NY, USA).

### RNA interference

p53 siRNA, PIG3 siRNA and negative control siRNA were purchased from Ambion (Austin, TX). The cells were transfected with 5 nmol/L of specific or negative control siRNA using INTERFERin siRNA transfection reagent according to the manufacturer’s instructions (Polyplus-Transfection, Inc., New York, NY). 24 h post-transfection, the cells were used for subsequent experiments.

### Assay for catalase activity

Cells were sonicated in 0.1 M Tris-HCl (pH7.5) for two 30-s bursts. After centrifugation, the supernatant was used to measure catalase activity with an Amplex Red catalase assay kit, according to the manufacturer’s protocol (Molecular Probes). After incubating the samples with 40 mM H_2_O_2_ for 30 min, the remaining H_2_O_2_ was measured to determine the catalase activity. Amplex Red and horseradish peroxidase react with H_2_O_2_ to produce resorufin, a fluorescent compound detectable by spectrophotometry. Standard curves for the enzymatic activity of catalase were determined using purified catalase. The protein concentration was measured using a BCA Protein Quantitation Analysis Kit (Solarbio). The enzyme activities were normalized by protein content of samples and expressed relative to the value in the control group.

### Animal study

6 to 7 week old C57BL/6 wild-type and p53 knockout mice were obtained from Vital River, (Beijing, China). Animal Care and procedures were approved by the Institutional Animal Care and Use Committee (China Agricultural University). The experiments were carried out in accordance with the approved guidelines. For the time-course experiment, patulin (2.5 mg/kg, dissolved in saline) was given by intraperitoneal (i.p.) injection for 1, 3, 6 or 12 h and then the samples were collected for analysis of the oxidative stress biomarkers; for the comparison experiment between p53 wild-type and knockout mice, 20 wild-type mice (WT, p53+/+) and 20 p53 knockout mice (p53-KO, p53−/−) were randomly assigned to four groups (each n = 10): WT Control, WT patulin, p53-KO Control, p53-KO patulin. All groups were treated with i.p. injection of saline or patulin (2.5 mg/kg, dissolved in saline).The animals were sacrificed 3 hours after the patulin administration. Kidney tissues were collected and frozen immediately in liquid nitrogen and stored at −80 °C.

### Histopathology assessment

Frozen kidney sections were stained with 10 μmol/L H_2_DCFDA for 30 min at 37 °C. Cells staining positively for the oxidized dye were identified by confocal microscopy.

### Biochemical assay

Kidney tissue was homogenized in saline to produce a 10% homogenate. Homogenates were centrifuged and the supernatant was collected for the measurement of LPO, GSH level and CAT activities, which were determined spectrophotometrically using test kits (Nanjing Jiancheng Institute of Biotechnology, Nanjing, China), according to the manufacturer’s instructions.

### Statistical analysis

Data are presented as mean ± SD. These data were analyzed by one-way ANOVA with appropriate post-hoc comparisons among means. *p* < 0.05 was considered statistically significant.

## Additional Information

**How to cite this article**: Jin, H. *et al.* p53 activation contributes to patulin-induced nephrotoxicity via modulation of reactive oxygen species generation. *Sci. Rep.* **6**, 24455; doi: 10.1038/srep24455 (2016).

## Supplementary Material

Supplementary Information
