# Supplementary material for: p53 activation contributes to patulin-induced nephrotoxicity via modulation of reactive oxygen species generation
Source: Sci Rep. 2016 Apr 13;6:24455. doi: 10.1038/srep24455 (PMC4829895; doi:10.1038/srep24455)
Supplement: Supplementary Information [file srep24455-s1.pdf]

Supplementary information:

p53 activation contributes to patulin-induced nephrotoxicity via modulation of reactive oxygen species generation

Huan Jin, Shutao Yin, Xinhua Song, Enxiang Zhang, Lihong Fan and Hongbo Hu

FigS1

A.

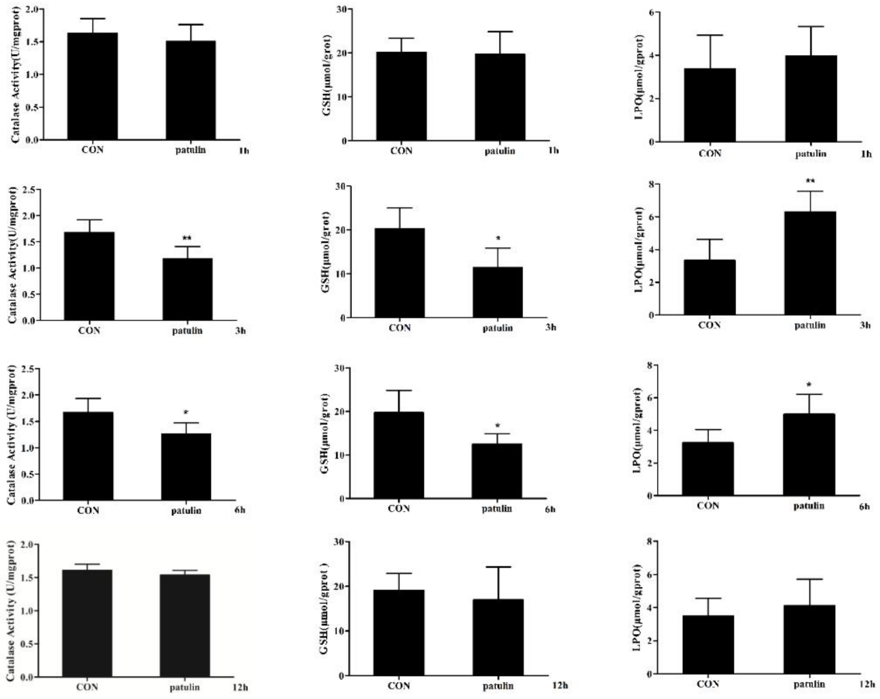

A.

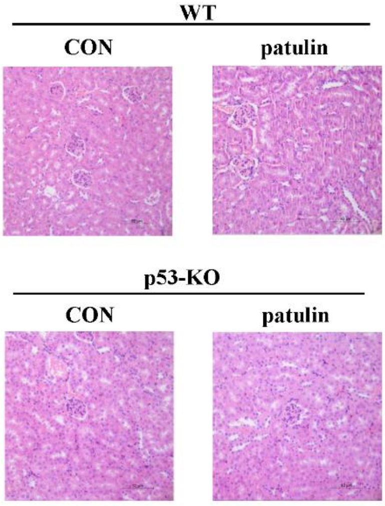

C.

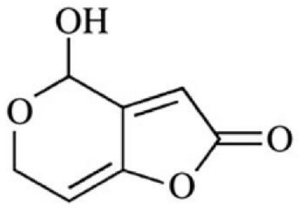

Figure S1 A. The kinetic process of patulin-induced oxidative stress in vivo. Patulin (2.5mg/kg, dissolved in saline) was given by i.p injection for 1, 3, 6 or 12 h and then the samples were collected for analysis of the oxidative stress biomarkers. B. Histopathological changes of kidney tissues by patulin in p53wide type/knockout mice measured by H&E staining. C. The chemical structure of patulin.
